# IgA and IgG antibody detection of mycobacterial antigens in pleural fluid and serum from pleural tuberculous patients

**DOI:** 10.1186/s12865-019-0315-y

**Published:** 2019-10-17

**Authors:** Renan Jeremias da Silva, Raquel da Silva Corrêa, Isabela Gama Sardella, Ana Carla de Paulo Mulinari, Thiago Thomaz Mafort, Ana Paula Santos, Rogério Rufino, Luciana Silva Rodrigues, Maria Helena Féres Saad

**Affiliations:** 1 Laboratorio de Microbiologia Celular, Instituto Oswaldo Cruz (IOC), Fundação Oswaldo Cruz (FIOCRUZ), Av. Brazil, 4365, Rio de Janeiro, 21040-360 Brazil; 2 Laboratório de Immunopatologia (LIP), Faculdade de Ciências Médicas, Universidade do Estado do Rio de Janeiro (UERJ), Rio de Janeiro, RJ Brazil; 3 Serviço de Pneumologia e Tisiologia, Hospital Universitário Pedro Ernesto (HUPE)/UERJ, Rio de Janeiro, RJ Brazil

**Keywords:** *Mycobacterium tuberculosis*, Pleural tuberculosis, IgA, IgG, ELISA, ADA

## Abstract

**Background:**

A previous study demonstrated pleural fluid (PF) IgA immunodominance for the fused MT10.3:MPT64 protein in pleural tuberculosis (PLTB) cases. However, no clue on the role of IgA and IgG against this and other antigens in PF and serum concerning improved diagnosis is available. Thus, the aim of the present study was to validate PF IgA-MT10.3:MPT64 and evaluate PF and serum IgA and IgG reactivity against this protein, its peptides (F2) and single MPT64, MT10.3 and the PPE59 mycobacterial specific antigens. IgA and IgG ELISA were measured against the antigen in PLTB (*n* = 29) and other non-TB pleurisy (*n* = 39) patient samples.

**Results:**

The immunodominance of PF IgA-MT10.3:MPT64 was confirmed in PLTB (86.2%) followed by PPE59 (62%), while serum IgA-F2 exhibited 51.7% sensitivity. PF and serum IgG-MT10.3:MPT64 led to 65.5 and 51.7% sensitivity, respectively. However, MT10.3 and MPT64 displayed overall lower sensitivity (≤34.5) for both antibodies. All results at 95% fixed specificity. Combinatory results indicated 93.1% sensitivity for PF IgA-MT10.3:MPT64/−PPE59 and IgA/IgG-MT10.3:MPT64 at 92.3% specificity, followed by IgA-MT10.3:MPT64/−MPT64 or /−F2 (89.6%) without jeopardizing specificity (94.9%). The combinatory results of the PF adenosine deaminase test (ADA) and IgA-MT10.3:MPT64/−F2 demonstrated the highest sensitivity (96.6%), with a specificity of 92.3%.

**Conclusions:**

The PF IgA-MT10:MPT64 immune dominance was validated in PLTB, and its combinatory results with PPE59 or MPT64 or F2 antigens as well as with IgG, are reported herein for the first time, improving their potential to assist diagnosis. Combining PF-ADA and IgA-MT10.3:MPT64/−F2 results achieved better accuracy. Moreover, serum IgG, although less accurate, displays potential beyond microbiological tests.

## Background

Pulmonary tuberculosis is the most common clinical manifestation of *Mycobacterium tuberculosis* (MTB) infection. Among extrapulmonary presentations pleural tuberculosis (PLTB) is the most frequent in many countries, representing 15% of the extrapulmonary cases globally reported in 2016 [1–3]. Moreover, PLTB is the major cause of pleural effusions, responsible for approximately 50% of all related diagnoses in Brazil [4, 5]. The disease generally affects immunocompetent young adults, but, although patients may spontaneously heal, there is a risk of developing active tuberculosis (TB) in the absence of specific drug administration. Thus, early diagnosis and adequate treatment are required to avoid PLTB evolution to tuberculous empyema or pleural fibrosis [6, 7].

Microbiological methods depend on the presence of bacilli in clinical specimens, therefore displaying low sensitivity in paucibacilary cases, like PLTB patients. The use of molecular techniques is costly and restricted to centers with adequate structure and specialized professionals. Histopathological examination of pleural biopsy samples comprises the standard PLTB reference test, but exhibits variable sensitivity, is expensive, time-consuming, requires skilled personnel and an invasive procedure [8, 9]. Serological tests based on antibodies (Ab) response may be an attractive alternative as a diagnostic method, as they are simple, fast and easy to operate, allowing their application in the public health system, where the diagnostic demand is high. In addition, they are not dependent on the presence of the bacilli in clinical specimens. In a previous study, Araujo et al. [10] demonstrated that the MT10.3:MPT64 fused antigen is recognized by immunoglobulin A (IgA) in the pleural fluid (PF) of PLTB patients, at high sensitivity (81.4%) and specificity (95.5%). Furthermore, the cloned fusion protein based on MPT64 and MT10.3 epitopes, termed F2 (MT10.3_(1M-40S)_:MPT64m_(91L-205A)_:MT10.3_(41S-96)_), was described to be predominantly recognized by serum IgA in pulmonary TB [11]. The PE (Pro-Glu) and PPE (Pro-Pro-Glu) molecules are coding by two families of genes responsible for about 10% of the MTB genome [12]. Proteins belonging to PPE family are involved in many aspects of TB pathogenesis, including antibody recognition and bacilli persistence ability in granulomas [13–16]. Moreover, their antigenic variation has been associated with the ability of determined microbial pathogens to evade the immune system [17]. The PPE59 protein coding gene *rv3429*c belonged the RD11, region absent in *M. bovis.* The actual role of PPE59 in metabolism or in the clinical evolution of MTB infection is not well known, but it is capable of inducing a cell-mediated response by IFN-γ and interleukin-10 [13, 15]. However, no clues on the reactivity to these antigens (Ags) by Ab of PF and/or serum of PLTB cases are available.

On the other hand, the use of adenosine deaminase (ADA) biochemical test may contribute to a more efficient and differential diagnosis in PLTB cases [1, 18–20]. Although it is a fast, cheap, reproducible and an easy to perform assay [8, 9], it presents several limitations: the predictive value depends on the local TB prevalence, low sensitivity in immunocompromised patients, false-positive results due to cross-reactivity with other diseases presenting pleural effusion, such as lymphoma, vascular collagen diseases and bacterial empyema. Furthermore, due a wide range of results from region to region should be used with caution in countries with low TB incidence [21–23]. Therefore, identification of new biomarkers and, consequently, the development of diagnostic test that differentiate PLTB from other pathologies presenting exudative pleural effusion may have a significant impact on primary care. In this context, here we investigated potential PLTB biomarkers, validating and/or evaluating the accuracy of dosing IgA and IgG response to mycobacterial single Ags MT10.3, MPT64 and PPE59, fusion proteins MT10.3:MPT64 and the novel F2, in PF and sera from patients with pleural effusion using an in-house ELISA method.

## Results

### Participant characteristics

**Fig. 1:**
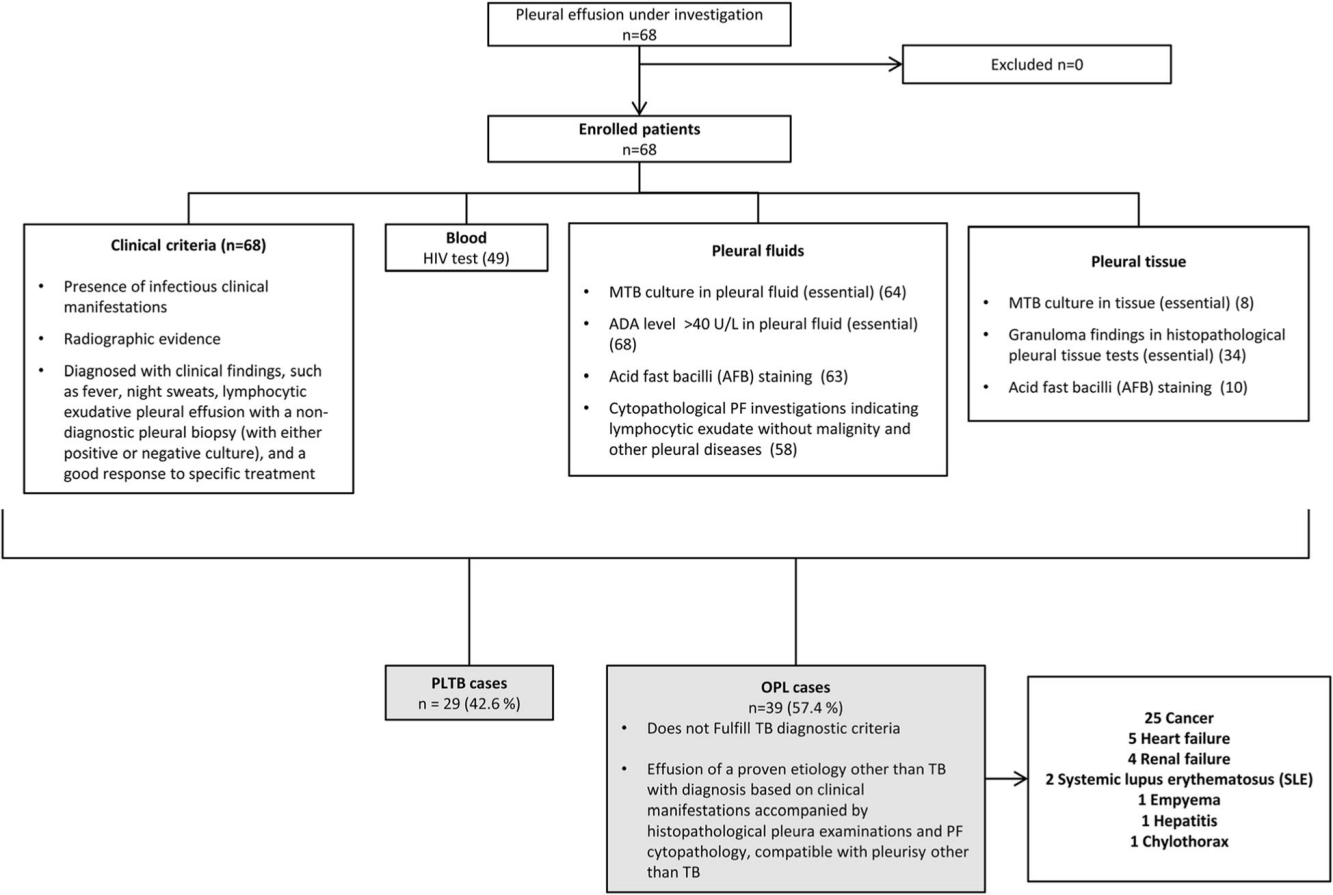
Flow chart of the study design, classification of study participants and diagnostic testing performed. Composite reference standard (CRS) used for categorization of study participants. AFB: acid-fast bacilli; ADA: adenosine deaminase; PLTB: Pleural tuberculosis. Round blankets: numbers refer to the patients submitted to the correspondent diagnostic test. Gray boxes show the final diagnosis and the study groups

Sixty-eight subjects with pleural effusion were enrolled in this study. As the microbiological testes yielded a low sensitivity and a universal composite reference standard (CRS) for PLTB diagnosis is not available, a CRS based on the clinical history, microbiological, radiological, and biochemical findings associated with PLTB based on recently published literature [24] was used by HUPE health professionals for final diagnosis [25]. The patients were classified into 2 categories, 29 (42.6%) PLTB and 39 (57.4%) with other non-tuberculous pleurisy (OPL) (Fig. 1).

**Fig. 2:**
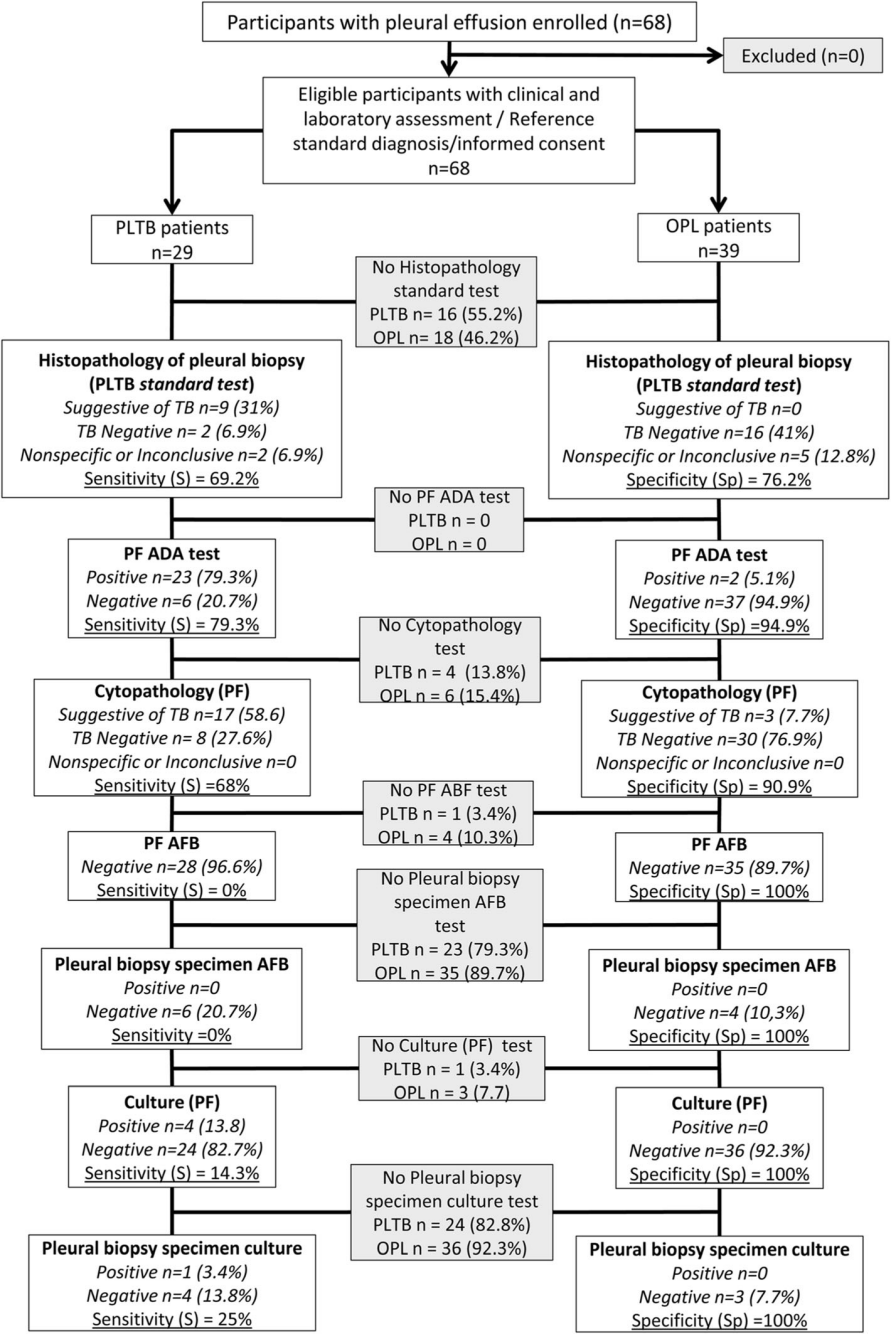
Standards for reporting of diagnostic accuracy (STARD) flow diagram showing the conventional microbiological, histopathological and adenosine deaminase (ADA) tests determined in pleural fluid (PF) and/or pleural biopsy (PB) samples for tuberculous pleural infection (PLTB) diagnosis

The main clinical, laboratory, and epidemiological data are depicted in Additional file 1: Table S1. Subjects differ in age, as OPL patients were older (*p* < 0.001) and the cancer pathology was dominant (64.1%), male gender was the majority of PLTB (72.5%), although no significant difference compared to OPL was observed (*p* = 0.12). Few participants reported past TB treatment (4/68, 5.8%), including two OPL patients. Concerning patients whose samples underwent conventional and ADA diagnostic tests, the PF-ADA investigation elicited adequate sensitivity and high specificity (79.3%, 23/29 and 94.9%, 37/39), followed by pleural biopsy histopathology suggestive of TB (69.2%, 9/13 and 76.2%, 16/21), respectively. Lower sensitivity and high specificity were obtained for the pleural biopsy (25%, 1/5 and 100%, 3/3) and PF culture (14.3%, 4/28 and 100%, 36/36) and acid-fast bacilli (AFB) smears (0%, 0/28 and 100%, 35/35), respectively (Fig. 2).

### Test results

#### Pleural fluid and serum IgA and IgG antibodies against MT10.3:MPT64, F2, PPE59, MT10.3 and MPT64

**Fig. 3:**
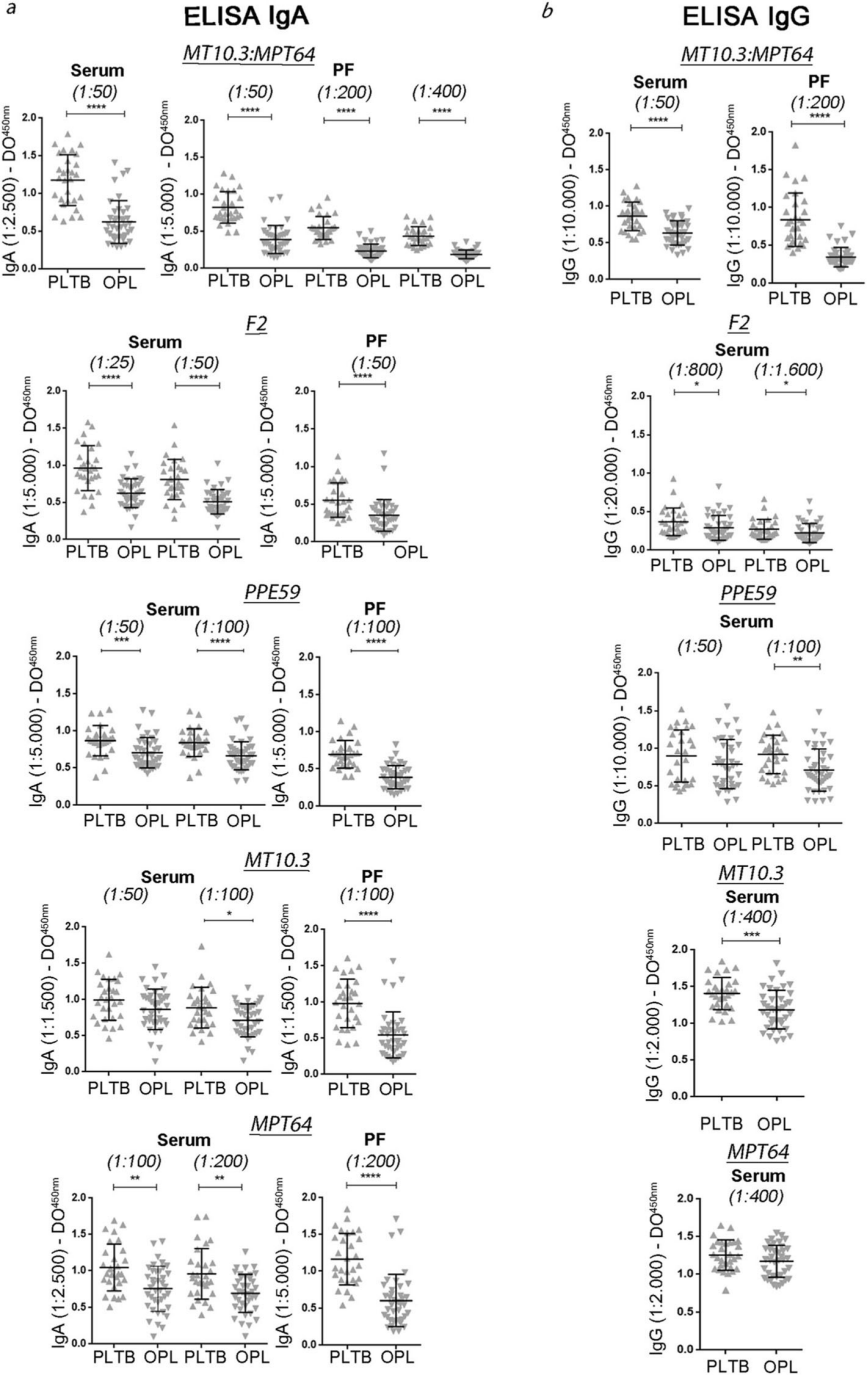
Distribution of individual humoral responses of (**a**) IgA and (**b**) IgG antibodies against different mycobacterial Ag, MT10.3, MPT64, PPE59, the MT10.3:MPT64 and the F2 fusion protein, in different dilutions of pleural fluid (PF) and serum of patients with pleural tuberculosis (PLTB) or other pleurisy non-TB (OPL). Note: Short bars, mean ODs. ****, *p* ≤ 0.0001,;***, *p* ≤ 0.0009; **, *p* ≤ < 0.001; *, *p* ≤ 0.05

**Fig. 4:**
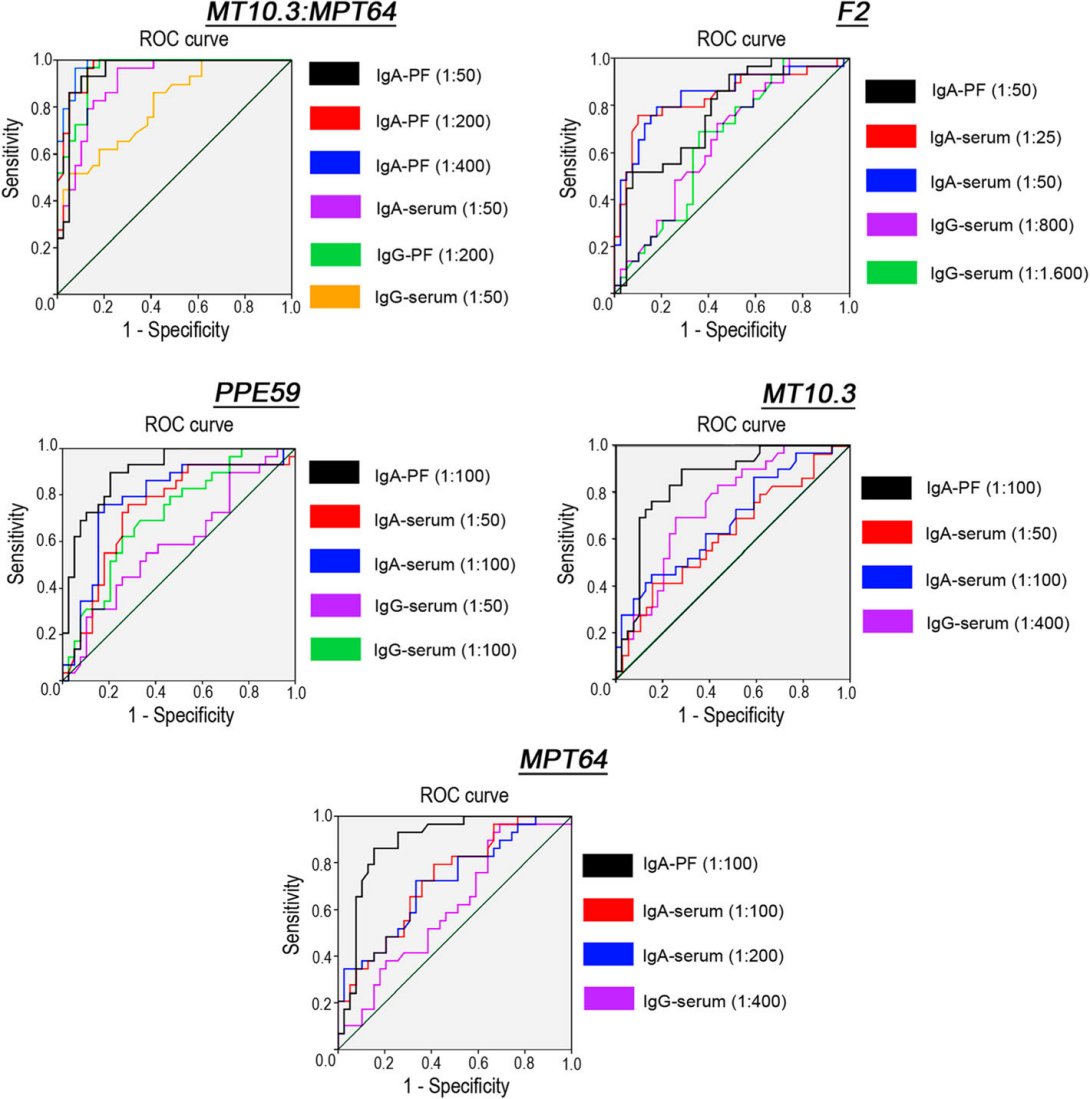
ROC curve of the IgA and IgG antibodies against different mycobacterial Ag, MT10.3, MPT64, PPE59, the MT10.3:MPT64 and the F2 fusion proteins, at different dilutions of pleural fluid (PF) and serum of patients with pleural tuberculosis (PLTB) or other pleurisy non-TB (OPL)

All enrolled participants had ELISA serum and PF examined for specific Ab in response to the five proposed antigens. The significantly high mean levels in PLTB compared to OPL for all Ab and antigens (*p* ≤ 0.031), except IgG (MPT64, PPE59 and F2) and IgA (MT10.3), at different sera dilutions (*p* ≥ 0.057), are displayed in Fig. 3a and b. Different samples dilutions were considered to evaluate whether results from different dilutions favored sensitivity without altering the specificity of the ELISA test. According to the receiver operating characteristic (ROC) curve analysis, when fixing specificity at 95%, a high positivity of PF IgA-MT10.3:MPT64 was confirmed registering the best sensitivities at 1:50 and 1:200 specimen dilutions (86.2%), followed by 82.8%, at 1:400. PF IgA-PPE59 (62.1%) and -F2 (44.8%) elicited moderate and low sensitivities at 1:100 and 1:50 dilutions, respectively. All tests showed AUC > 0.904, except for F2. Serum IgA-F2 reached the best positivity (51.7%) at both 1:25 and 1:50 dilutions and AUC < 0.844. For IgG, the only antigen recognized by PLTB patients with high AUC (0.952) was the MT10.3:MPT64, with sensitivity of 65.5% in PF at a 1:200 dilution, decreasing to 51.7% in serum at a 1:50 dilution and AUC = 0.809 (Table 1 and Fig. 4).

**Table 1:** ELISA validity parameters for the detection of IgA and IgG antibodies against mycobacterial antigens MT10.3, MPT64, PPE59, and the MT10.3:MPT64 and the F2 fusion proteins, at different pleural fluid (PF) and serum dilutions

ELISA tests (samples dilutions)	PL-TB (*n* = 29)	OPL (*n* = 39)	p valor^a^	Cut off	AUC	Sensitivity (%) (Number of +)	Specificity (%) (Number of -)
	Mean ± SD	Mean ± SD					
**IgA-(PF)**							
MT10.3 (1:100)	0.983 ± 0.336	0.548 ± 0.321	*	1.278	0.852	20.7 (6)	95 (37)
MPT64 (1:100)	1.164 ± 0.350	0.606 ± 0.352	*	1.505	0.887	24.1 (7)	95 (37)
**MT10.3-MPT64 (1:50)**	0.821 ± 0.213	0.387 ± 0.188	*	0.658	0.949	**86.2 (25)**	95 (37)
MT10.3-MPT64 (1:100)	0.667 ± 0.195	0.302 ± 0.138	*	0.598	0.950	65.5 (19)	95 (37)
MT10.3-MPT64 (1:200)	0.542 ± 0.154	0.232 ± 0.093	*	0.385	0.968	86.2 (25)	95 (37)
MT10.3-MPT64 (1:400)	0.433 ± 0.126	0.185 ± 0.060	*	0.315	0.980	82.8(24)	95 (37)
MT10.3-MPT64 (1:800)	0.323 ± 0.125	0.209 ± 0.094	*	0.404	0.823	27.6 (8)	95 (37)
**PPE59 (1:100)**	0.696 ± 0.186	0.392 ± 0.155	*	0.639	0.904	**62.1 (18)**	95 (37)
F2 (1:50)	0.553 ± 0.230	0.351 ± 0.212	*	0.542	0.774	44.8 (13)	95 (37)
**IgA-(serum)**							
MT10.3 (1:50)	0.996 ± 0.282	0.865 ± 0.279	0.081	1.282	0.625	20.7 (6)	95 (37)
MT10.3 (1:100)	0.888 ± 0.281	0.714 ± 0.228	0.012	1.018	0.678	27.6 (8)	95 (37)
MT10.3 (1:200)	0.760 ± 0.263	0.634 ± 0.209	0.057	0.962	0.636	13.8 (4)	95 (37)
MT10.3 (1:400)	0.622 ± 0.216	0.528 ± 0.172	0.130	0.799	0.608	13.8 (4)	95 (37)
MPT64 (1:50)	1.109 ± 0.341	0.843 ± 0.371	0.003	1.462	0.708	20.7 (6)	95 (37)
MPT64 (1:100)	1.045 ± 0.323	0.757 ± 0.308	*	1.220	0.729	27.6 (8)	95 (37)
MPT64 (1:200)	0.959 ± 0.347	0.693 ± 0.258	0.002	1.048	0.717	34.5 (10)	95 (37)
MPT64 (1:400)	0.860 ± 0.338	0.630 ± 0.231	0.006	1.012	0.695	27.6 (8)	95 (37)
MT10.3:MPT64 (1:50)	1.176 ± 0.338	0.621 ± 0.282	*	1.267	0.905	44.8 (13)	95 (37)
PPE59 (1:50)	0.868 ± 0.204	0.706 ± 0.206	*	1.042	0.736	13.8 (4)	95 (37)
PPE59 (1:100)	0.841 ± 0.189	0.663 ± 0.190	*	1.043	0.785	13.8 (4)	95 (37)
**F2 (1:25)**	0.963 ± 0.304	0.624 ± 0.195	*	0,957	0.837	**51.7 (15)**	95 (37)
**F2 (1:50)**	0.810 ± 0.271	0.507 ± 0.165	*	0,772	0.844	**51.7 (15)**	95 (37)
F2 (1:100)	0.698 ± 0.282	0.414 ± 0.134	*	0,670	0.847	48.3 (14)	95 (37)
F2 (1:200)	0.619 ± 0.252	0.443 ± 0.188	*	0,591	0.879	44.8 (13)	95 (37)
**IgG-(PF)**							
**MT10.3-MPT64 (1:200)**	0.840 ± 0.352	0.347 ± 0.126	*	0.616	0.952	**65.5 (19)**	95 (37)
**IgG-(serum)**							
**MT10.3:MPT64 (1:50)**	0.863 ± 0.196	0.636 ± 0.195	*	0.891	0.809	**51.7 (15)**	95 (37)
MT10.3 (1:400)	1.409 ± 0.218	1.190 ± 0.261	*	1.641	0.739	17.2 (5)	95 (37)
MPT64 (1:400)	1.256 ± 0.201	1.173 ± 0.213	0.129	1.490	0.609	10.3 (3)	95 (37)
PPE59 (1:50)	0.918 ± 0.252	0.789 ± 0.327	0.204	1.370	0.591	3.4 (1)	95 (37)
PPE59 (1:100)	0.898 ± 0.346	0.709 ± 0.279	0.003	1.181	0.711	17.2 (5)	95 (37)
F2 (1:200)	0.604 ± 0.319	0.499 ± 0.313	0.087	1.084	0.622	6.9 (2)	95 (37)
F2 (1400)	0.467 ± 0.267	0.387 ± 0.232	0.127	0.787	0.609	10.3 (3)	95 (37)
F2 (1:800)	0.369 ± 0.180	0.289 ± 0.161	0.024	0.552	0.660	13.8 (4)	95 (37)
F2 (1:1600)	0.271 ± 0.127	0.223 ± 0.123	0.031	0.427	0.663	10.3 (3)	95 (37)

IgA and IgG mean level results in optical density and their standard deviations (SD)

*PLTB* pleural tuberculosis patients, *OPL* patients with other, non-TB, pleural diseases, *AUC* area under the curve

^a^Significant difference of IgA/IgG production between groups and between tested antigen were calculated by the Mann Whitney test (*p* < 0.001*)

Boldface: represents the antigens tests of best sensitivities

It is noteworthy that, when combining the PF IgA-MT10.3:MPT64 (1:50) -MPT64 (1:100) or -F2 (1:50), or IgA-MT10.3:MPT64/−PPE59 (1:200/1:100, respectively) results, sensitivity improved to 89.6% without jeopardizing specificity (94.9%). These results were not obtained for any other tested Ags, although PF IgA-MT10.3:MPT64/−PPE59 (1:50/1:100) and IgA/IgG- MT10.3:MPT64 combinatory results elicited the highest sensitivity (93.1%), indicating a slight decrease in specificity (92.3%), while serum IgG (1:50/1:800 or 1:1600) led to the best combination result (55.1%) for both fusion proteins MT10.3:MPT64/F2, with the same specificity (Table 2). Other IgA and/or IgG antigen combinatory results have reached moderate to high sensitivities (> 68%), however, jeopardizing specificity (89.7%).

**Table 2:** IgA and/or IgG MT10.3:MPT64 Pleural Fluid (PF) and/or sera dilutions combination results with other antigens or ADA results that provide the best sensibility (S) and specificity (Sp) for pleural tuberculosis patients (PLTB)

MT10.3:MPT64 Combination results (sample dilutions)/	PLTB (*N* = 29) N. Positive S (%)	OPL (*N* = 39) N. Negative Sp (%)
Pleural Fluid IgA		
(1:50) / PPE59 (1:100)	27 (93.1)	36 (92.3)
(1:100)/ PPE59 (1: 100)	24 (82.7)	37 (94.8)
**(1:200) / PPE59 (1:100);**	**26** (**89.6**)	**37** (**94.8**)
**(1:50) / MPT64 (1:100);**		
**(1:50) / F2 (1:50)**		
(1:400)/ PPE59 (1:100);	26 (89.6)	36 (92.3)
(1:50) or /(1:200) or /(1:400) / MT10.3 (1:100)		
(1:800) / PPE59 (1:100);	21 (72.4)	36 (92.3)
(1:100) / MT10.3 (1:100)		
(1:100) / MPT64 (1:100)	20 (68.9)	36 (92.3)
(1:200) / MPT64 (1:100) or / F2 (1:50) or / MT10.3:MPT64.serum1.50	25 (86.2)	36 (92.3)
(1:400) / MPT64 (1:100) or / F2 (1:50)	24 (82.7)	36 (92.3)
(1:100) / F2 (1:50)	22 (75.8)	36 (92.3)
(1:50) / MT10.3-MPT64.serum1.50	26 (93.1)	35 (89.7)
(1:1000) / MT10.3-MPT64.serum1.50	19 (65.5)	36 (92.3)
Serum IgA		
(1.50) / MPT64 (1:50)	13 (44.8)	36 (92.3)
(1.50) / MPT64 (1:200)	14 (48.2)	36 (92.3)
Serum IgG		
(1:50) / F2 (1:400)	15 (51.7)	36 (92.3)
(1:50) / F2 (1:800 or 1:1600)	16 (55.1)	36 (92.3)
Pleural Fluid IgA/IgG		
(1:50)/ MT10.3:MPT64 (1:200)	27 (93.1)	36 (92.3)
Pleural Fluid IgA		
(1:50) / F2 (1:50)/ ADA	28 (96.6%)	36 (92.3%)

*OPL* other non-tuberculous pleurisy

Boldface: represents the combinatory test results of best sensitivities and specificity

**Fig. 5:**
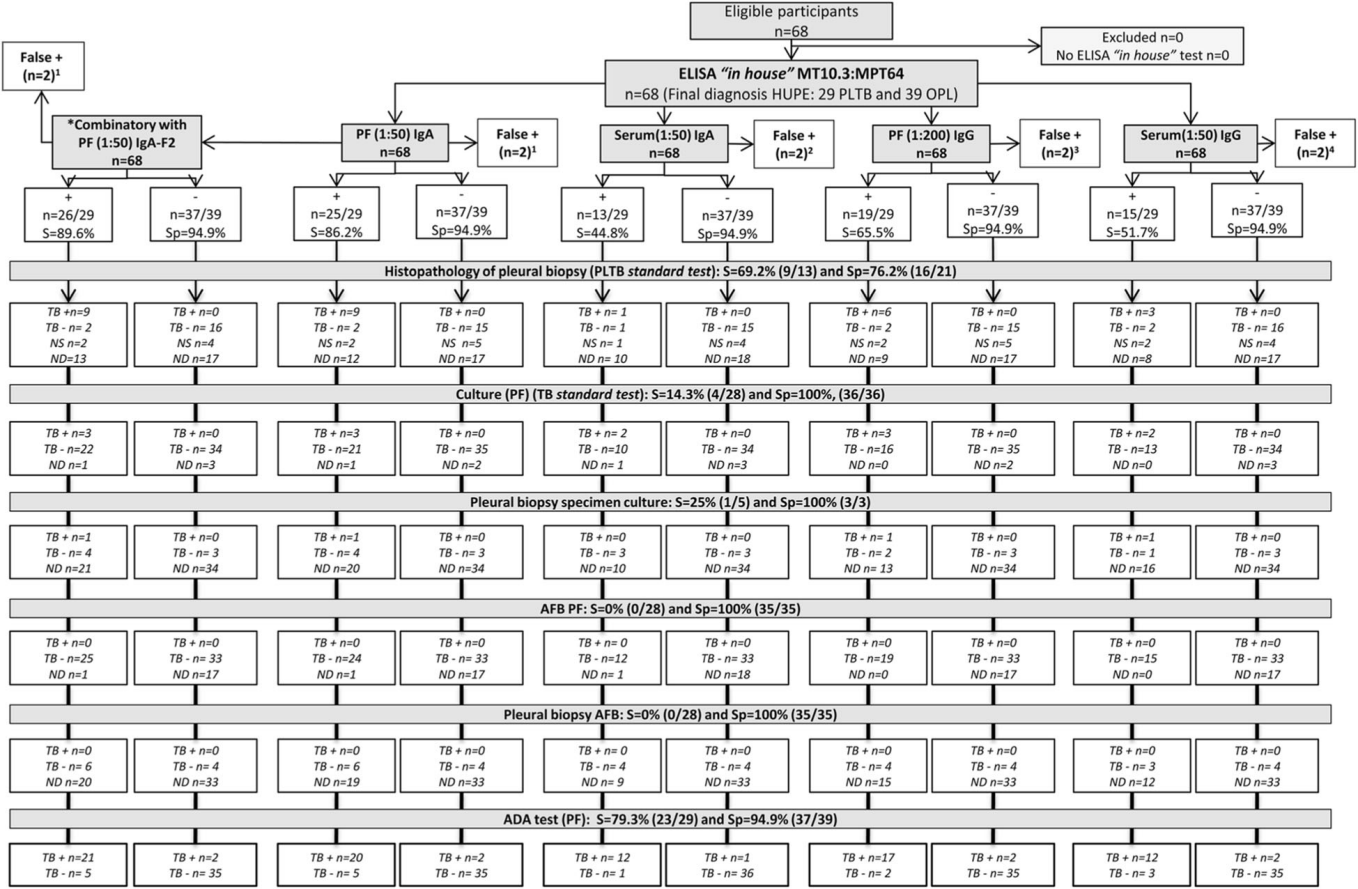
Flow diagram (according to STARD (Standards for the Reporting of Diagnostic accuracy)) of the best ELISA results compared with conventional microbiological, histopathological and adenosine deaminase (ADA) tests determined in pleural fluid (PF) and/or pleural biopsy (PB) samples for tuberculous pleural infection (PLTB) diagnosis. Note: *combinatory results of pleural fluid IgA-MT10.3-MPT64 diluted 1:50 with -MPT64 (1:100) or –F2 (1:50). OPL: other non-TB pleurisies. ^1^ Patient with cancer (*n* = 1), patient with heart failure (*n* = 1), PF ADA and AFB staining tests negative, without histopathological examination; ^2^ Patient with cancer (*n* = 1), PF ADA and pleural biopsy histopathology negative results, patient with lupus (*n* = 1), PF ADA positive, PF AFB and culture tests negative, nonspecific histopathological examination; ^3^ Patient with cancer (*n* = 2), both PF ADA negative results; 1 PF AFB and culture tests negative, nonspecific histopathological examination and another without PF ABF and histopathological examination; ^4^ Patient with cancer (*n* = 2), both PF ADA, AFB and culture tests negatives; nonspecific histopathological examination (*n* = 1) or without histopathological examination(*n* = 1). + = positive; − = negative; NS = Nonspecific, ND = no data (teste was not performed)

The cross reactivity was observed in only two OPL patient for PF at 1:50 dilution associated to both, IgA-MT10.3:MPT64 and IgA-F2 (Fig. 5), being diagnosed with cancer and heart failure, respectively. The patient with cancer presented negative results for PF-ADA and pleural biopsy histopathology. The patient with heart failure showed negative results for PF-ADA and AFB staining tests, histopathological examination was not done. However, one this patient tested positive for the QuantiFERON®-TB Gold In-Tube (QFT-GIT) assay (data not shown). These results may suggest latent tuberculosis infection (LTBI).

#### Comparison of standard conventional tests and ADA assay with the ELISA in pleural tuberculosis diagnoses

Considering the best results for PF or serum IgG and IgA with different antigens, we hypothesized if they were more accurate in comparison with other tests used to diagnosis PLTB. As depicted in Fig. 5, PF-ADA (S = 79.3% and Sp = 94.9%) or pleural biopsy histopathology (S = 69.2%, 9/13 and Sp = 76.2%, 16/21) were less accurate than PF IgA-MT10.3:MPT64 (1:50) and its combinatory results with /MPT64 (1:100) or /F2 (1:50), which elicited S ≥ 89.6% and Sp = 94.9%. Thus, PF-IgA ELISA produced better positivity to that conventional testing, but the ELISA positivity among negative results provided by those conventional tests in PLTB is noteworthy. Although histopathologic examination was not done for all patients, 15.3% were non-specific but positively identified by the ELISA, which positively also detected PLTB cases without known histopathological exam (12/16, 75%). Thus, the ELISA resulted in an overall sensitivity of 86.2%, against 69.2% of the histopathological exam. On the other hand, most of the PLTB cases with negative ADA resulted in positive PF IgA-MT10.3:MPT64 diluted at 1:50 (5/6), as well as their combinatory results with /MPT64 (1:100) or /F2 (1:50) (5/6, respectively). Moreover, microbiological tests also not done in all samples, performed weaker (> 25%), as expected in paucibacilary cases, such as PLTB. However, the ELISA proposed herein provided significantly better detection (ranging from 51.7 to 89.6%) for IgG or IgA determined in serum (51.7% or 44.8%) or PF (65.5% or > 86%, respectively), especially for the combinatory PF (1:50) IgA-MT10.3:MPT64/F2 results (Table 2, Fig. 5). None of the other combinatory results led to better accuracy.

One of the disadvantages of the immune humoral test is the Ab response heterogeneity across patients [26–28]. As the present study evaluated a small number of antigens or their fused peptides, some of the patients did not respond. Thus, we evaluate the ELISA combined with ADA results, as both tests were performed for all samples, aiming at diagnostic improvement. The combined results of PF (1:50) IgA-MT10.3-MPT64/−F2 and ADA provided the best sensitivity (96.6%, 28/29) for PLTB detection, at 92.3% specificity (36/39) (Table 2).

## Discussion

Simple, fast and accurate diagnostic methods in PLTB are still required. For instance, the reference histopathological examination for pleural biopsy was virtually not performed in the present sampling as compared to Araújo et al [10]. On the contrary, the diagnosis herein relied on clinical suspicion and the simple ADA test for all patients, reaching 79% sensitivity. However, PF IgA MT10.3:MPT64 (86.2%) and it combined results with /−MPT64 or /−F2 identified all, except one, PLTB negative ADA, and, despite missing three or two of the positive ADA PLTB patients, the ELISA improved pleurisy tuberculosis detection (89.6%). The combinatory results for PF (1:50) IgA MT10.3:MPT64/−F2 and ADA displays a double advantage, as, besides increasing sensitivity (96.6%), the PF can be used at the same dilution for both fused Ags when compared to the combinatory results of different PF dilutions used to obtain the same high sensitivity, thus making them suitable as a diagnosis test to aid in PLTB diagnosis. Moreover, the combinations of tests results can obviate the need for a pleural biopsy during the initial diagnostic and, as the ELISA and ADA are fast techniques and of low costs, easy to operate and to apply in public health systems, they favor a rapid auxiliary PLTB diagnosis. Recently, authors have suggested the use of ADA in combination with other diagnostic tests, such as interferon-gamma (IFN-γ) release assays, and in the search for new biomarkers [18–20, 29–32]. The present study provides a simpler alternative ELISA for ADA combination.

Notably, the better fused antigen MT10.3:MPT64 performance for PF IgA detection corroborates previous studies [10], although the lower reactivity on single antigens MT10.3 (20.7%) and MPT64 (24.1%) was not expected and is in disagreement with the previous report (72%) assessing populations carrying similar pleurisy morbidities to the present study [33]. One explanation could be antigen degradation, as the batches used herein were old and underwent several freezing and thawing cycles. On the other hand, for serum, the present results corroborate those reported by Silva et al. [34] where, even evaluating pulmonary TB, both single antigens failed to show high sensitivity (< 34.5%). Nevertheless, reduced recognition of these antigens by serum IgA and IgG (including to PPE59) was also detected.

It is well known that IgA is a predominant mucosal/serous immunoglobulin, also secreted in the pleural space [35, 36]. However, IgA against mycobacterial Ags has been found in pulmonary TB patient sera by several authors [11, 37–40]. Zhao et al. [41] suggested Ab-based tests in plasma to identify Beijing MTB infection. In our study, IgG and IgA resulted in lower serum positivity compared to PF results, but the frequency of positive results is higher to that in microbiological tests, therefore useful in their absence and other, less sensitive, conventional PLTB diagnostic tests. The use of recombinant fused antigens in diagnostic tests constitutes an interesting strategy to increase accuracy and simplicity. However, fusion of different antigens may change reactivity according to gene position in their construction [10, 11, 42]. The higher sensitivity elicited by MT10.3:MPT64 may be related to this fact, since the fusion protein expressed by opposite genes according to that construction seems to display low stability and quality (personal communication).

As expected, histopathological examinations demonstrated higher sensitivity than the conventional microbiological methods for PLTB diagnosis, but lower compared to the developed ELISA. Nonetheless, among the patients with negative results or no information, the ELISA PF IgA-MT10.3:MPT64 was able to identify most PLTB patients, which could be supportive of the hypothesis that, in some cases, pleural effusion may also be caused by the possible entry of mycobacterial antigens into the pleural space, thereby stimulating the presence of IgA at this site [43]. Conversely, few OPL patients diagnosed by histopathology presented false-positive ELISA IgA-MT10.3:MPT64 results (1/16, 6.2%).

The F2 (MT10.3_(1M-40S)_:MPT64m_(91L-205A)_:MT10.3_(41S-96)_) containing peptides of both antigens led to decreased mean levels of reactivity to almost half among PLTB cases (0.553 ± 0.230), but no change for OPL cases (0.351 ± 0.212) compared to the full fused genes (0.821 ± 0.213 or 0.387 ± 0.188, respectively). However, F2 recognition by a PLTB group added sensitivity to the combinatory of results using both fusion antigens for PF IgA detection. This may be explained by exposure of other epitopes not available in the full gene construction or single antigens.

Until now, no publications associating the F2 protein chimera or PPE59 in PF IgA detection in PLTB patient are available. Despite their lower sensitivity compared to MT10.3:MPT64, sensitivity was higher to that AFB smear exams. Among microbiological tests used for TB diagnosis, AFB is the flagship, as it is easy to apply, rapid and cheap, although exhibiting low sensitivity, nevertheless considering that the ELISA has the potential to be point of care (POC) test, similar results to those found in bacterioscopy assessments may be obtained quickly. Serum IgA-F2 elicited the best reactivity compared to the other antigens, detecting half of the PLTB cases (51.7%); although in pulmonary TB this positivity was evidenced for IgG–F2 [11], a discrepancy which may be related to the different clinical TB forms. It is possible that the compliance of the fused protein, where the repositioning of the gene fragments led to the formation of new epitopes and, consequently, obfuscation of others, could positively affect IgA or IgG recognition in PL or pulmonary TB patient sera. The third single antigen used in this study, PPE59, performed similarly, at low reactivity for serum IgG in PLTB, as previously observed for pulmonary and different extrapulmonary TB cases (25 and 0%, respectively) (unpublished data). However, a higher PF (1:100) IgA-PPE59 (62.1%) sensitivity compared to that unpublished data (54 and 28%, respectively) is described herein for the first time. To date, PPE59 it is known to induce cell-mediated response by IFN-γ and interleukin-10 in pulmonary TB [12, 15] and, as a result of our study, immunodominance of PF IgA but non-IgG in PLTB patient. Therefore, PPE59 protein displays potential as specific TB diagnosis markers and may assist in the diagnostic investigations performed in PLTB.

Some Bacille Calmette Guérin (BCG) vaccine strains, including the one used in Brazil, contain the gene coding for the MPT64 antigen and it has been hypothesized that tumor cells and the BCG strain may share antigens [10, 44, 45]. In the present study, cross-reactivity was observed, as in previous similar studies [10, 26, 46, 47]. In our and in the other similar Brazilian [10] studies the majority of participants were BCG vaccinated and, more important, they live in a city with a high TB incidence rate [48, 49]. Thus, it is possible the OPL patients may have presented TB as undiagnosed co-morbidity or were LTBI, since some of them presented QFT-GIT® positive results. Lupus autoimmune disorder based diagnostics rely solely on clinical examination, negative PF culture or AFB and positive for the ADA assay. Another possible explanation is that, 64% of OPL cases presented cancer and higher mean IgA levels in both PF and serum, as well as PF IgG, compared to other OPL cases. False-positive ELISA results in patients with lung cancer have been reported by other authors [33, 35].

This study has some limitations, such as: 1) in the absence of a precise diagnostic tests for PLTB and the limitations of the ADA, it is possible that patients were in some stage of TB status, including LTBI, but were mistakenly classified as OPL because not all the resources available for the TB diagnosis were applied, 2) patients recruitment was performed in a single healthcare center, which contributes to the limitation in the number of the cases included, 3) failure to follow the clinical evolution of the patients, which would make it possible to ascertain TB disease in false-positive cases in the tests proposed here.

## Conclusions

A successful ELISA for the detection of IgA and IgG in body fluids of relative invasive power, such as PF and serum against different mycobacterial antigens for PLTB diagnosis was described. In addition, the MT10:MPT64 fusion protein was validated as highly promising marker in a new PLTB cohort, although presenting less sensitivity for IgG in serum, both with high specificity diagnostic potential. PPE59 as a single antigen recognized by PF and serum IgA at high sensibility and specificity compared to conventional microbiological assays was described for the first time. The combinatory results of PF (1:50) IgA MT-10:MPT64/−F2 performed better than conventional diagnostic tests, but their combination with ADA improved PLTB diagnostic, which may contribute to the adequate treatment to avoid PLTB evolution to tuberculous emphysema or pleural fibrosis. Finally, it is underway the development of a PF IgA- MT10.3:MPT64, −PPE59 or IgA MT-10:MPT64/F2 rapid diagnostic tool based on lateral flow as prove of concept.

## Methods

### Study design, participants and ethical statement

Observational, cross-sectional and prospective study where sera and PF samples from 68 individuals attended by spontaneous demand and diagnosed with PLTB or OPL at the Hospital Universitário Pedro Ernesto (HUPE)/UERJ, were collected from March 2015 to March 2017. The inclusion criterion was the presence of pleural effusion with clinical indication for thoracentesis, as described elsewhere [33]. All participants were ≥ 18 years old. Exclusion criteria were, pregnant women, those who did not agree to participate or provide clinical specimens and those who refused to sign the informed consent form. The study population included: **i)** PLTB cases, presence of infectious clinical manifestations accompanied by ADA positivity (> 40 U/L), and/or positive acid-fast bacilli (AFB) staining and/or positive MTB culture in pleural fluid or tissue; and/or by granuloma findings in histopathological pleural tissue tests; and/or cytopathological PF investigations indicating lymphocytic exudate without malignity; and/or diagnosed with clinical findings, such as fever, night sweats, lymphocytic exudative pleural effusion with a non-diagnostic pleural biopsy (with either positive or negative culture), and a good response to specific treatment; and **ii)** OPL cases, with diagnosis based on clinical manifestations accompanied by histopathological pleura examinations and PF cytopathology, compatible with pleurisy other than TB. Most patients presented information regarding BCG vaccinations (62/68). PF samples underwent the ADA test based on Giusti’s method [50], at the Hermes Pardini Laboratory. Human immunodeficiency virus (HIV) infection status was determined in sera using the Cobas®, chemiluminescence test (Roche Molecular Systems Inc., CA, USA). Pleural tissue for biopsies and PF samples underwent AFB staining and culture in Lowenstein-Jensen’s (LJ) medium, according to routine laboratory methods. Pleural biopsy fragments were taken for histopathological examinations and PF, for cytopathological investigations. This study was approved by the HUPE/UERJ Ethics Committee, no. 1.100.772. Sample donation was obtained only after written informed consent of all participants. Clinical, laboratory, and demographic data were obtained from medical records.

### Test methods

#### Clinical specimens and sample processing

Venous blood was collected in 10 mL vacutainer tubes (BD Biosciences, Mountain View, CA) and PF was collected in 50 mL conical Falcon tubes at the time of diagnosis, and immediately processed. Samples were centrifuged at 930 x g for 10 min, at 25 °C for serum or 4 °C for PF. Supernatant aliquots were then stored at − 80 °C until use. PF or sera samples with notable hemolysis were excluded from the study. All procedures follow international biosecurity standards.

#### Antigens

The recombinant fused antigens MT10.3:MPT64, F2 (MT10.3_(1M-40S)_:MPT64m_(91L-205A)_:MT10.3_(41S-96)_), and PPE59 were engineered in our laboratory, as described elsewhere [10, 11, 16]. LIONEX Diagnostics & Therapeutics GmbH (Braunschweig, Germany) kindly donated Ags MT10.3 and MPT64.

#### IgA and IgG “*in house* “enzyme immunosorbent assay (ELISA)

Flat-bottom polystyrene microtiter plates (Maxisorp; Nunc, Swedesboro, NJ, USA) were coated and incubated for 2 h at 37 °C with 50 μL/well of an antigen solution in carbonate-bicarbonate buffer (CBB, pH 9.6), at concentrations previously determined. The plates were washed three times with 200 μL of phosphate buffered saline with 0.01% tween 20 (PBSt) pH = 7.4 and blocked by adding 100 μL/well of 5% bovine serum albumin in PBSt (BSA-PBSt) and incubated, as described above. After washing, the plates were immediately used or stored at 4 °C (> 2 weeks). Each 50 μL of serial 2-fold dilutions of PF or sera samples in 1% BSA-PBSt were added to the respective wells and incubated for 1 h at 37 °C. After being washed, new incubation on 50 μL solution of horseradish peroxidase-conjugated goat anti-human IgA or IgG monoclonal antibodies (HRP, Pierce, Rockford, IL) at different dilutions in 1% BSA-PBSt was used. After a final washing, HRP activity was detected adding 50 μL/well of the chromogenic 3.3,5.5-tetramethylbenzidine substrate solution (TMB; Pierce, Rockford, IL), and leaving the plate for 20 min in the dark at room temperature. To stop the reactions, 50 μL/well of 2 mol. L^− 1^ sulfuric acid was added and the absorbance was read at 450 nm. All tests were performed in duplicate, and, pooled positive and negative controls (dilution previously determined) were used as references in each set of experiments. To estimate IgG-MT10.3:MPT64 immunoreactivity, the same procedure was used, with minor modification. The washings, dilutions of the samples and conjugates carried out, respectively, with PBSt 0.1%, and PBSt 0.1% plus 0.3 mol. L^− 1^ NaCl as suggested by Araujo et al. [10]. An Additional file 2: Table S2 file exhibits the antigen, sample and conjugate standardizations and parameterizations for the ELISA tests.

### Statistical analyses

*M. tuberculosis* protein immunoreactivity was determined by optical density (OD). Results are expressed as means ± standard deviation (SD), and groups were compared using the Mann-Whitney or Kruskal-Wallis test. The chosen cutoff value was calculated for each antigen according to a receiver operating characteristic (ROC) curve analysis for each PF and serum dilution, fixing specificity at 95%, based on all PLTB and OPL subjects. The diagnostic value of the ELISA was evaluated in terms of sensitivity (S) and specificity (Sp). A *p* value (*p*) of ≤0.05 was considered statistically significant. A combinatory analysis of the results of the different proteins with test results used for routine diagnosis was carried out. Statistical analyses were performed using the *Statistical Package for Social Sciences* (SPSS) v. 20 (SPSS Inc., Chicago, IL) and Prism6 (Graph-Pad Software Inc., San Diego, CA) software.

## Supplementary information

**Additional file 1:** **Table S1.** Clinical and socioeconomic data of participants presenting pleural tuberculosis (PLTB) or non-TB pleural diseases (OPL). Supplementary Table S1 depicts the main clinical, laboratory, and epidemiological data of participants enrolled in this study. Note this table: * *p* < 0.05. SD - Standard Deviation.1 ADA – adenosine deaminase cutoff of > 40 U/L, PL-TB: pleural tuberculosis patients; OPL: patients with others non-TB pleural diseases. SLE: Systemic lupus erythematosus. Note: In Brazil, pardo (brown) means a mixture of European, Black and Amerindian.

**Additional file 2:** **Table S2.** The standardizations and parameterizations of antigens concentration, samples and conjugates dilutions for the IgA and IgG ELISA detection in pleural fluid (PF) and serum of pleural tuberculosis (PLTB) and other pleurisy non TB (OPL). An Supplementary Table S1 exhibits the antigen, sample and conjugate standardizations and parameterizations for the ELISA tests. Note this table: X: represent the ELISA not possible parameters in the standardization.

## Data Availability

The dataset that support the findings of this study are available from the authors but restrictions apply to the availability of these data, which were used under license for the current study and so are not publicly available. Data are however available from the corresponding author upon reasonable request.

## Abbreviations

Ab: Antibody
ADA: Adenosine deaminase
AFB: Acid-Fast Bacilli stain
Ags: Antigens
BCG: Bacille Calmette Guérin
BSA: Bovine serum albumin
C: graus Celsius
CBB: Carbonate-bicarbonate buffer
CNPq: Conselho Nacional de Desenvolvimento Científico e Tecnológico
ELISA: Enzyme-liked ImunoSorbent Assay
F2: protein chimera MT10.3(1 M-40S):MPT64M(91 L-205A):MT10.3(41S-96))
FAPERJ: Fundação de Amparo à Pesquisa do Estado do Rio de Janeiro
FIOCRUZ: Oswaldo Cruz institution
h: hours
HIV: Human immunodeficiency virus
HUPE: Hospital Universitário Pedro Ernesto
IFN: Interferon-gamma
IgA: Immunoglobulin A
IgG: Immunoglobulin G
LIP: Laboratório de Imunopatologia
LJ: Lowenstein-Jensen’s medium
LTBI: Latent tuberculosis infection
ml: milliliter
MPT64 (Rv1980c): MTB antigen (24 kDa) encoded by genes of the difference region 2
MT10.3 (ES6.9, TB10.3, Rv3019c): MTB antigen (10 kDa) encoded by genes of the difference region 2
MT10.3:MPT64: MTB antigens fusion MT10.3 and MPT64
MTB: *Mycobacterium tuberculosis*
NaCl: sodium chloride
nm: nanometer
OD: Optical density
OPL: Other non-tuberculous pleurisy
p: p valor
PBSt: Phosphatebuffered saline with tween
PF: Pleural fluid
PLTB: Pleural tuberculosis
POC: Point of care test
PPE59: MTB antigens encoded by RD11. PE (Pro-Glu) e PPE (Pro-Pro-Glu) Family
Prism6: Prism Graph-Pad Software
QFT-GIT: QuantiFERON®-TB Gold In-Tube assay
RJ: Rio de Janeiro
ROC: Receiver operating characteristic
S: Sensitivity
SD: Standard deviations
Sp: Specificity
SPSS: Statistical Package for Social Sciences software
TB: Tuberculosis
TMB: 3,3,5,5-tetramethylbenzidine substrate solution
UERJ: Universidade Estadual do Rio de Janeiro
x g: g Force
μl: microliter
