# Supplementary material for: IgA and IgG antibody detection of mycobacterial antigens in pleural fluid and serum from pleural tuberculous patients
Source: BMC Immunol. 2019 Oct 17;20:36. doi: 10.1186/s12865-019-0315-y (PMC6798396; doi:10.1186/s12865-019-0315-y)
Supplement: Supplementary file 1 — Additional file 1: Table S1. Clinical and socioeconomic data of participants presenting pleural tuberculosis (PLTB) or non-TB pleural diseases (OPL). Supplementary Table S1 depicts the main clinical, laboratory, and epidemiological data of participants enrolled in this study. Note this table: * p < 0.05. SD - Standard Deviation.1 ADA – adenosine deaminase cutoff of > 40 U/L, PL-TB: pleural tuberculosis patients; OPL: patients with others non-TB pleural diseases. SLE: Systemic lupus erythematosus. Note: In Brazil, pardo (brown) means a mixture of European, Black and Amerindian. [file 12865_2019_315_MOESM1_ESM.docx]

Supplementary Table S1 – Clinical and socioeconomic data of participants presenting pleural tuberculosis (PLTB) or non-TB pleural diseases (OPL).

| Variables | PLTB  n=29 (42.6%) | OPL  n=39 (57.4%) |
| --- | --- | --- |
| *Age* (mean ± SD) | 43.07±16.59 | 62.5±18.63 * |
| < 50 years | 22 (75.9) | 10 (25.6) |
| > 50 years | 7 (24.1) | 29 (74.4) |
| *Gender* |  |  |
| Male | 21 (72.5) | 21 (53.8) |
| *Ethnicity* |  |  |
| White | 6 (20.7) | 18 (46.2) |
| Black | 6 (20.7) | 10 (25.6) |
| Pardo (Brown) | 17 (58.6) | 11 (28.2) |
| *BCG vaccination:* |  |  |
| Positive (scar) | 20 (69) | 20 (51.3) |
| No Information | 1 (3.4) | 5 (12.8) |
| *ADA (mean ± SD) ^1^* | 61.15±32.71 * | 19.54±31.29 |
| Positive | 23 (79.3) | 2 (5.1) |
| *Previous TB* |  |  |
| Positive | 2 (6.9) | 2 (5.1) |
| No Information | 0 | 2 (5.1) |
| *Other morbidity* |  |  |
| Cancer | 0 | 25 (64.1) |
| Heart failure | 0 | 5 (12.8) |
| Renal failure | 0 | 4 (10.3) |
| SLE | 0 | 2 (5.1) |
| Hepatitis, Empyema, Chylothorax | 0 | 3 (7.7) |
| *Previous TB treatment* |  |  |
| Free | 28 (96.6) | 37 (94.9) |
| >1 month | 1 (3,4) | 2 (5.1) |
| *Human immunodeficiency virus (HIV) status* |  |  |
| Positive | 1 (3.4) | 2 (5.1) |
| No Information | 5 (17.3) | 14 (35.9) |

* p<0.05. SD - Standard Deviation.1 ADA – adenosine deaminase cutoff of > 40 U/L, PL-TB: pleural tuberculosis patients; OPL: patients with others non-TB pleural diseases. SLE: Systemic lupus erythematosus. Note: In Brazil, pardo (brown) means a mixture of European, Black and Amerindian.
