# Supplementary material for: IgA and IgG antibody detection of mycobacterial antigens in pleural fluid and serum from pleural tuberculous patients
Source: BMC Immunol. 2019 Oct 17;20:36. doi: 10.1186/s12865-019-0315-y (PMC6798396; doi:10.1186/s12865-019-0315-y)
Supplement: Supplementary file 2 — Additional file 2: Table S2. The standardizations and parameterizations of antigens concentration, samples and conjugates dilutions for the IgA and IgG ELISA detection in pleural fluid (PF) and serum of pleural tuberculosis (PLTB) and other pleurisy non TB (OPL). An Supplementary Table S1 exhibits the antigen, sample and conjugate standardizations and parameterizations for the ELISA tests. Note this table: X: represent the ELISA not possible parameters in the standardization. [file 12865_2019_315_MOESM2_ESM.docx]

Supplementary Table S2 - The standardizations and parameterizations of antigens concentration, samples and conjugates dilutions for the IgA and IgG ELISA detection in pleural fluid (PF) and serum of pleural tuberculosis (PLTB) and other pleurisy non TB (OPL).

|  | IgA | IgG | Standardization |
| --- | --- | --- | --- |
| PPE59 (µg/mL)  Serum (dilution)  Secondary antibody | 0,5  1:50 and 1:100  1:2.500 | 1,0  1:50 and 1:100  1:10.000 | Mulinari, 2016 |
| PPE59 (µg/mL)  PF (dilution)  Secondary antibody | 1,0  1:100  1:5.000 | X | present study |
| F2 (µg/mL)  Serum (dilution)  Secondary antibody | 0,5  1:25 to 1:200  1:5.000 | 1,5  1:200 to 1:1600  1:20.000 | Sardella *et al*., 2014 |
| F2 (µg/mL)  PF (dilution)  Secondary antibody | 1,5  1:50  1:5.000 | X | present study |
| MT10.3:MPT64 (µg/mL)  Serum (dilution)  Secondary antibody | 0.05  1:50  1:2.500 | 1,0  1:50  1:10.000 | present study |
| MT10.3:MPT64 (µg/mL)  PF (dilution)  Secondary antibody | 0,5  1:50 to 1:800  1:5.000 | 1,0  1:200  1:10.000 | Araújo *et al*., 2010 (IgA);  present study (IgG) |
| MT10.3 (µg/mL)  Serum (dilution)  Secondary antibody | 1,0  1:50 a 1:400  1:1.500 | 1,0  1:400  1:2.000 | Silva *et al*. 2008 |
| MT10.3 (µg/mL)  PF (dilution)  Secondary antibody | 1,0  1:100  1:5.000 | X | Kaisermann *et al*., 2005 |
| MPT64 (µg/mL)  Serum (dilution)  Secondary antibody | 0,5  1:50 a 1:400  1:2.500 | 1,0  1:400  1.2.000 | Silva *et al*. 2008 |
| MPT64 (µg/mL)  PF (dilution)  Secondary antibody | 1,0  1:100  1:5.000 | X | Kaisermann *et al*., 2005 |

X: represent the ELISA not possible parameters in the standardization
